# On Satnoianu-Wu's Inequality

**DOI:** 10.1155/2013/645201

**Published:** 2013-07-16

**Authors:** Bo-Yan Xi

**Affiliations:** College of Mathematics, Inner Mongolia University for Nationalities, Tongliao City, Inner Mongolia 028043, China

## Abstract

By applying techniques in the theory of convex functions and Schur-geometrically convex functions, the author investigates a conjecture of Satnoianu on an algebraic inequality and generalizes some known results in recent years.

## 1. Introduction

For *n* ∈ *ℕ* = {1,2, 3,…} and *x*_*i*_ ∈ ℝ_+_ = (0, *∞*) with *i* = 1,2,…, *n*, let

(1) $$\begin{array}{c} A_{n}\left(x\right)=\frac{1}{n}\sum_{i=1}^{n}x_{i},\;\;G_{n}\left(x\right)={\left(\prod_{i=1}^{n}x_{i}\right)}^{1/n},\\ H_{n}\left(x\right)=\frac{n}{\sum_{i=1}^{n}\left(1/x_{i}\right)}. \end{array}$$

These quantities are, respectively, called the arithmetic, geometric, and harmonic means of a positive sequence *x* = (*x*_1_, *x*_2_,…, *x*_*n*_). For more information on the theory of means, see the monograph [1] or the papers [2–5] and plenty of references therein. 

 For convenience, in what follows, we use the notation *x* ^*q*^ = (*x*_1_^*q*^, *x*_2_^*q*^,…, *x*_*n*_^*q*^) for

(2) $$\begin{array}{c} 0<m\leq x_{1}^{q},x_{2}^{q},\ldots ,x_{n}^{q}\leq M, \end{array}$$

and *q* ∈ ℝ = (−*∞*, *∞*).

 In [6], Satnoianu posed the following conjecture.

**Conjecture 1**

For *n* ≥ 2, *x*_*i*_ > 0, and *λ* ≥ *n* ^*n*−1^ − 1, it is valid that

(3) $$\begin{array}{c} \sum_{i=1}^{n}{\left(\frac{x_{i}^{n-1}}{x_{i}^{n-1}+\lambda \prod_{k=1,k\neq i}^{n}x_{k}}\right)}^{1/(n-1)}\geq \frac{n}{{\left(1+\lambda \right)}^{1/(n-1)}}. \end{array}$$

This conjecture has been solved and researched in [7–10]. Among them, Wu obtained in [10] the following result.

**Theorem 2 (see [10, Theorem 1])**

Let *n* be a positive integer and let *α*, *β*, and *x*_*i*_ for *i* = 1,2,…, *n* be positive numbers. If *p* ≠ 0 and

(4) $$\begin{array}{c} \beta \geq \left(n^{\max\{p,1\}}-1\right)\alpha , \end{array}$$

then

(5) $$\begin{array}{c} \sum_{i=1}^{n}{\left[\frac{x_{i}^{q}}{\alpha x_{i}^{q}+\beta G\left(x^{q}\right)}\right]}^{1/\text{p}}\geq \frac{n}{{\left(\alpha +\beta \right)}^{1/p}}. \end{array}$$

It is clear that inequality (5) generalizes (3). Therefore, we would like to call inequality (5) Satnoianu-Wu's inequality.

Now we naturally pose the following questions:

1. Can one improve the condition (4)?
2. Does the reversed inequality of (5) exist?
3. Are there other types of inequalities of Satnoianu-Wu type?

The goal of this paper is to answer these questions.

## 2. Definitions and Lemmas

We need the following definitions and lemmas.

**Definition 3 (see [11, page 8])**

Let *x* = (*x*_1_, *x*_2_,…, *x*_*n*_) ∈ ℝ^*n*^ and *y* = (*y*_1_, *y*_2_,…, *y*_*n*_) ∈ ℝ^*n*^. We say that *x* is majorized by *y* (in symbols *x*≺*y*) if

(6) $$\begin{array}{c} \sum_{i=1}^{k}x_{\left[i\right]}\leq \sum_{i=1}^{k}y_{\left[i\right]},\;\;\sum_{i=1}^{n}x_{i}=\sum_{i=1}^{n}y_{i}, \end{array}$$

for *k* = 1,2,…, *n* − 1, where *x*_[1]_ ≥ ⋯≥*x*_[*n*]_ and *y*_[1]_ ≥ ⋯≥*y*_[*n*]_ are rearrangements of *x* and *y* in a descending order.

**Definition 4 (see [12])**

Let *Ω*⊆ℝ_+_^*n*^.

1. The set *Ω* is said to be geometrically convex if (*x*_1_^*λ*^ *y*_1_^1−*λ*^,…, *x*_*n*_^*λ*^ *y*_*n*_^1−*λ*^) ∈ *Ω* for every *x*, *y* ∈ *Ω* and *λ* ∈ [0,1].
2. A function *φ* : *Ω* → ℝ_+_ is said to be Schur-geometrically convex on *Ω* if ln⁡*x* = (ln⁡*x*_1_,…, ln⁡*x*_*n*_)≺ln⁡*y* = (ln⁡*y*_1_,…, ln⁡*y*_*n*_) implies *φ*(*x*) ≤ *φ*(*y*) for every *x*, *y* ∈ *Ω*.
3. A function *φ* : *Ω* → ℝ_+_ is said to be Schur-geometrically concave on *Ω* if ln⁡*x* = (ln⁡*x*_1_,…, ln⁡*x*_*n*_)≺ln⁡*y* = (ln⁡*y*_1_,…, ln⁡*y*_*n*_) implies *φ*(*x*) ≥ *φ*(*y*) for every *x*, *y* ∈ *Ω*.

**Lemma 5 (see [12])**

Let *Ω*⊆ℝ_+_^*n*^ be a symmetric and geometrically convex set with inner points and *φ* : *Ω* → ℝ_+_ a symmetric and differentiable function in *Ω*°. Then *φ* is a Schur-geometrically convex (or Schur-geometrically concave, resp.) function on *Ω* if and only if

(7) (ln⁡x1−ln⁡x2)[x1∂φ(x)∂x1−x2∂φ(x)∂x2]≥0     (or≤0, resp.), x∈Ω∘.

**Lemma 6 (see [1, page 4, Bernoulli's inequality])**

The inequality

(8) $$\begin{array}{c} {\left(1+t\right)}^{r}\geq 1+rt \end{array}$$

holds for *r* ≥ 1 and *t* ≥ −1 or for *r* ≤ 0 and *t* > −1. If  0 < *r* < 1 and *t* ≥ −1, inequality (8) is reversed.

## 3. Main Results

Now we start off to demonstrate our main results.

**Theorem**

Let *m*, *M* be defined as in (2) and *n* ≥ 2, let *α*, *β*, *x*_*i*_ ∈ ℝ_+_ for *i* = 1,2,…, *n*, and let *p*, *q* ∈ ℝ with *p* ≠ 0. If (1 − *p*)*mβ* ≥ 2*pMα*, then

(9) $$\begin{array}{c} \sum_{i=1}^{n}{\left[\frac{x_{i}^{q}}{\alpha x_{i}^{q}+\beta A_{n}\left(x^{q}\right)}\right]}^{1/p}\geq \frac{n}{{\left(\alpha +\beta \right)}^{1/p}}. \end{array}$$

If (1 − *p*)*Mβ* ≤ 2*pmα*, inequality (9) is reversed.

**Proof**

For *M*′ > *m*′ > 0 and *r* ∈ ℝ with *r* ≠ 0, since

(10) $$\begin{array}{c} {\left[{\left(\frac{t}{1+t}\right)}^{r}\right]}^{\prime \prime }=\frac{r\left(r-1-2t\right)t^{r-1}}{{\left(1+t\right)}^{r+2}},\;t\in \left[m^{\prime },M^{\prime }\right], \end{array}$$

then

1. for *r* < 0, or for *r* > 1 and *r* − 1 − 2*M*′ > 0, the function (*t*/(1 + *t*))^*r*^ is convex on [*m*′, *M*′];
2. for 0 < *r* ≤ 1, or for *r* > 1 and *r* − 1 − 2*m*′ < 0, the function (*t*/(1 + *t*))^*r*^ is concave on [*m*′, *M*′].

By Jensen's inequality, when *r* < 0, or when *r* > 1 and *r* − 1 − 2*M*′ > 0, we have

(11) $$\begin{array}{c} \sum_{i=1}^{n}{\left(\frac{u_{i}}{1+u_{i}}\right)}^{r}\geq n{\left[\frac{A_{n}\left(u\right)}{1+A_{n}\left(u\right)}\right]}^{r}, \end{array}$$

where *u* = (*u*_1_, *u*_2_,…, *u*_*n*_)∈[*m*′, *M*′]^*n*^. When 0 < *r* ≤ 1, or when *r* > 1 and *r* − 1 − 2*m*′ < 0, inequality (11) is reversed.

Let *u*_*i*_ = *αx*_*i*_^*q*^/*βA*_*n*_(*x* ^*q*^) for *i* = 1,2,…, *n*, *r* = 1/*p*, *m*′ = *αm*/*βM*, and *M*′ = *αM*/*βm*. Then it is easy to show that *A*_*n*_(*u*) = *α*/*β*. Making use of (11), we obtain inequality (9). The proof of Theorem 7 is complete.

**Corollary 8**

Under the conditions of Theorem 7 and when *M* = (*n* − 1)*m*, if (1 − *p*)*β* > 2*p*(*n* − 1)*α*, then

(12) $$\begin{array}{c} \sum_{i=1}^{n}{\left[\frac{x_{i}^{q}}{\alpha x_{i}^{q}+\beta A_{n}\left(x^{q}\right)}\right]}^{1/p}\geq \frac{n}{{\left(\alpha +\beta \right)}^{1/p}}; \end{array}$$

if (1 − *p*)(*n* − 1)*β* < 2*pα*, inequality (12) is reversed.

**Corollary 9**

Let *n* ≥ 2, *α*, *β*, *x*_*i*_ ∈ ℝ_+_ for *i* = 1,2,…, *n*, and *p*, *q* ∈ ℝ. If *p* < 0, then

(13) $$\begin{array}{c} \sum_{i=1}^{n}{\left[\frac{x_{i}^{q}}{\alpha x_{i}^{q}+\beta A_{n}\left(x^{q}\right)}\right]}^{1/p}\geq \frac{n}{{\left(\alpha +\beta \right)}^{1/p}}; \end{array}$$

if *p* ≥ 1, inequality (13) is reversed.

**Proof**

This follows from Theorem 7 by considering that if *p* < 0, we have (1 − *p*)*mβ* > 2*pMα* and that if *p* ≥ 1, we have (1 − *p*)*Mβ* < 2*pmα*.

**Theorem 10**

Let *n* ≥ 2, *α*, *β*, *x*_*i*_ ∈ ℝ_+_ for  *i* = 1,2,…, *n*, *p*, *q* ∈ ℝ with *p* ≠ 0, and *m*, *M* defined by (2). If  *mβ* ≥ *pMα*, then

(14) $$\begin{array}{c} \sum_{i=1}^{n}{\left[\frac{x_{i}^{q}}{\alpha x_{i}^{q}+\beta G_{n}\left(x^{q}\right)}\right]}^{1/p}\geq \frac{n}{{\left(\alpha +\beta \right)}^{1/p}}; \end{array}$$

if *Mβ* ≤ *m* *pα*, inequality (14) is reversed.

**Proof**

For *M*′ > *m*′ > 0, let

(15) $$\begin{array}{c} f\left(u\right)=\sum_{i=1}^{n}{\left(1+u_{i}\right)}^{r},\;u\in {\left[m^{\prime },M^{\prime }\right]}^{n}. \end{array}$$

Then

(16) $$\begin{array}{c} \frac{\partial f\left(u\right)}{\partial u_{i}}=r{\left(1+u_{i}\right)}^{r-1},\\ \frac{\partial }{\partial u_{i}}\left[u_{i}\frac{\partial f\left(u\right)}{\partial u_{i}}\right]=r{\left(1+u_{i}\right)}^{r-2}\left(1+ru_{i}\right), \end{array}$$

for 1 ≤ *i* ≤ *n*. When *r* > 0, or when *r* < 0 and 1 + *rm*′ < 0, we have

(17) $$\begin{array}{c} \left(\ln u_{1}-\ln u_{2}\right)\left[u_{1}\frac{\partial f\left(u\right)}{\partial u_{1}}-u_{2}\frac{\partial f\left(u\right)}{\partial u_{2}}\right]\geq 0,\;u\in {\left[m^{\prime },M^{\prime }\right]}^{n}; \end{array}$$

when *r* < 0 and 1 + *rM*′ > 0, inequality (17) is reversed.

Using Lemma 5, we have the following conclusions:

1. if *r* > 0, or if *r* < 0 and 1 + *rm*′ < 0, the function *f*(*u*) is Schur-geometrically convex on [*m*′, *M*′]^*n*^;
2. if *r* < 0 and 1 + *rM*′ > 0, the function *f*(*u*) is Schur-geometrically concave on [*m*′, *M*′]^*n*^.

By the fact that

(18) (ln⁡Gn(u),ln⁡Gn(u),…,ln⁡Gn(u))  ≺(ln⁡u1,ln⁡u2,…,ln⁡un), u∈ℝ+n,

and by Definition 4, if *r* > 0, or if *r* < 0 and 1 + *rm*′ < 0, we have

(19) $$\begin{array}{c} \sum_{i=1}^{n}{\left(1+u_{i}\right)}^{r}\geq n{\left(1+G_{n}\left(u\right)\right)}^{r},\;u\in {\left[m^{\prime },M^{\prime }\right]}^{n}; \end{array}$$

if *r* < 0 and 1 + *rM*′ > 0, inequality (19) is reversed.

Letting *u*_*i*_ = *βG*_*n*_(*x* ^*q*^)/*αx*_*i*_^*q*^ for *i* = 1,2,…, *n* leads to *G*_*n*_(*u*) = *β*/*α*. Putting *r* = −1/*p*, *m*′ = *βm*/*αM* and *M*′ = *βM*/*αm* in inequality (19) results in inequality (14). The proof of Theorem 10 is complete.

**Corollary 11**

Under the conditions of Theorem 10 and when *M* = (*n* − 1)*m*, if *β* ≥ *p*(*n* − 1)*α*, then

(20) $$\begin{array}{c} \sum_{i=1}^{n}{\left[\frac{x_{i}^{q}}{\alpha x_{i}^{q}+\beta A_{n}\left(x^{q}\right)}\right]}^{1/p}\geq \frac{n}{{\left(\alpha +\beta \right)}^{1/p}}; \end{array}$$

if (*n* − 1)*β* ≤ *pα*, inequality (20) is reversed.

**Remark 12**

When *n* ≥ 2 and *p* > 1, from Lemma 6 and (4), it follows that

(21) $$\begin{array}{c} \beta \geq \left(n^{\max\left\{p,1\right\}}-1\right)\alpha >p\left(n-1\right)\alpha . \end{array}$$

**Theorem 13**

Let *n* ≥ 2, *α*, *β*, *x*_*i*_ ∈ ℝ_+_ for *i* = 1,2,…, *n*, and *p*, *q* ∈ ℝ with *p* ≠ 0. If *p* ≥ −1, then

(22) $$\begin{array}{c} \sum_{i=1}^{n}{\left[\frac{x_{i}^{q}}{\alpha x_{i}^{q}+\beta H_{n}(x^{q})}\right]}^{1/p}\geq \frac{n}{{\left(\alpha +\beta \right)}^{1/p}}; \end{array}$$

if *p* ≤ −1, inequality (22) is reversed.

**Proof**

Since (1 + *t*)^*r*^ is a convex (or concave, resp.) function on ℝ_+_ for *r* ≥ 1 or *r* < 0 (or for 0 < *r* ≤ 1, resp.), by Jensen's inequality, if *r* ≥ 1 or *r* < 0, we have

(23) $$\begin{array}{c} \sum_{i=1}^{n}{\left(1+u_{i}\right)}^{r}\geq n{\left(1+A_{n}\left(u\right)\right)}^{r},\;u\in {\mathbb{R}}_{+}^{n}; \end{array}$$

if 0 < *r* ≤ 1, inequality (23) is reversed.

Letting *u*_*i*_ = *βH*_*n*_(*x* ^*q*^)/*αx*_*i*_^*q*^ and *r* = −1/*p* shows *A*_*n*_(*u*) = *β*/*α*. Further from (23), we obtain inequality (22). The proof of Theorem 13 is complete.

**Remark 14**

It is clear that inequalities (9) and (22) both generalize inequality (3).

**Corollary 15**

Let *n* ≥ 2, *α*, *β*, *x*_*i*_ ∈ ℝ_+_ for *i* = 1,2,…, *n*, *p*, *q* ∈ ℝ with *p* ≠ 0, and *m*, *M* defined as in (2).

(1) When  −1 ≤ *p* < 0, one has
  (24) ∑i=1n[xiqαxiq+βAn(xq)]1/p‍≥∑i=1n[xiqαxiq+βGn(xq)]1/p≥∑i=1n[xiqαxiq+βHn(xq)]1/p≥n(α+β)1/p.
(2) When 0 < *p* < 1 and (1 − *p*)*mβ* > 2*pMα*, one has
  (25) ∑i=1n[xiqαxiq+βHn(xq)]1/p≥∑i=1n[xiqαxiq+βGn(xq)]1/p≥∑i=1n[xiqαxiq+βAn(xq)]1/p≥n(α+β)1/p.
(3) When *p* > 0 and *mβ* > *pMα*, one has
  (26) ∑i=1n[xiqαxiq+βHn(xq)]1/p≥∑i=1n[xiqαxiq+βGn(xq)]1/p≥n(α+β)1/p.
(4) When *p* > 0 and *Mβ* < *m* *pα*, one has
  (27) ∑i=1n[xiqαxiq+βAn(xq)]1/p≤∑i=1n[xiqαxiq+βGn(xq)]1/p≤n(α+β)1/p.

**Proof**

This follows from utilizing the well-known harmonic-geometric-arithmetic mean inequality

(28) $$\begin{array}{c} H_{n}\left(x\right)\leq G_{n}\left(x\right)\leq A_{n}\left(x\right) \end{array}$$

and Theorems 7 to 13.

**Corollary 16**

Under the conditions of Corollary 15 and when *M* = (*n* − 1)*m*,

(1) if  0 < *p* < 1 and (1 − *p*)*β* > 2*p*(*n* − 1)*α*, one has
  (29) ∑i=1n[xiqαxiq+βHn(xq)]1/p≥∑i=1n[xiqαxiq+βGn(xq)]1/p≥∑i=1n[xiqαxiq+βAn(xq)]1/p≥n(α+β)1/p;
(2) if *p* > 0 and *β* > *p*(*n* − 1)*α*, one has
  (30) ∑i=1n[xiqαxiq+βHn(xq)]1/p≥∑i=1n[xiqαxiq+βGn(xq)]1/p≥n(α+β)1/p;
(3) if *p* > 0 and (*n* − 1)*β* < *pα*, one has
  (31) ∑i=1n[xiqαxiq+βAn(xq)]1/p≤∑i=1n[xiqαxiq+βGn(xq)]1/p≤n(α+β)1/p.
